# Effectiveness of interventions on early initiation of breastfeeding in South Asia: a systematic review and meta-analysis of randomized controlled trials

**DOI:** 10.1186/s13006-025-00736-2

**Published:** 2025-05-27

**Authors:** M. A. Rifat, Mahashweta Chakrabarty, Syeda Saima Alam, Masum Ali, Syeda Sumaiya Nasrin, Plabon Sarkar, Aditya Singh, Sanjib Saha

**Affiliations:** 1 Department of Global Public Health, Karolinska Institutet, Stockholm, 17176 Sweden; 2 Banaras Hindu University, Varanasi, Uttar Pradesh 221005 India; 3 Department of Food Technology and Nutrition Science, Noakhali Science and Technology University, Noakhali, 3814 Bangladesh; 4 Nutrition and Health Science, Laney Graduate School, Emory University, Atlanta, 30322 USA; 5 United Nations Children’s Fund, Cox’s Bazar, 4700 Bangladesh; 6 Caritas Bangladesh, 2, Outer Circular Road, Shantibagh, Dhaka 1217 Bangladesh; 7 Health Economics Unit, Department of Clinical Sciences, Lund University, Lund, 22100 Sweden

**Keywords:** Breastfeeding, Early breastfeeding, Breastfeeding initiation, Newborn care, Perinatal care, South Asia, Meta-analysis

## Abstract

**Background:**

Early initiation of breastfeeding, defined as breastfeeding within one hour of birth, halves the risk of neonatal mortality, establishing it as a crucial outcome component in various interventions implemented across South Asian countries. However, the overall effect of these interventions remain unexamined. Therefore, this study seeks to address this knowledge gap by evaluating the overall effect of these interventions on maternal early initiation of breastfeeding practice.

**Methods:**

A systematic literature search was performed to identify randomised controlled trials conducted in South Asia focusing on early initiation of breastfeeding as an outcome variable. The interventions identified were categorized into behavioral, mobile health (mHealth), health system strengthening, and nutritional interventions. Random effects meta-analysis was conducted to estimate the pooled effect of interventions and effectiveness by intervention categories. Heterogeneity was explored by sub-group and meta-regression analyses. The risk of bias and strength of evidence were assessed by Cochrane’s RoB2 assessment tool and GRADE criteria, respectively.

**Results:**

We included 22 articles published, representing 19 unique interventions, from a pool of 2,524 screened records for review and narrative synthesis. Among these, 19 articles were eligible for meta-analysis. The pooled relative risk (RR) of early initiation of breastfeeding among mothers in the intervention groups, as compared to their counterparts, was 1.55 (95% CI: 1.24, 1.95; I^2^ = 99.56; *p* < 0.001). Interventions targeted health system strengthening represented stronger effect than other types of interventions. The overall strength of evidence was moderate.

**Conclusion:**

The overall intervention effect appeared efficacious in improving maternal early initiation of breastfeeding practice in South Asia, providing valuable insights for policymakers to develop contextually feasible strategies.

**Supplementary Information:**

The online version contains supplementary material available at 10.1186/s13006-025-00736-2.

## Background

Early initiation of breastfeeding, defined as breastfeeding within one hour of birth, ensures that the newborn receives colostrum, mother’s first milk, at the earliest possible after birth. Colostrum significantly contributes to the survival and wellbeing of children by providing them with all the essential nutrients and boosting their immunity, for which it is recognized as the first vaccine for a newborn [1, 2]. Early initiation of breastfeeding effectively halves the risk of infant mortality across all live births [3, 4] while also fostering exclusive breastfeeding, which yields long-term health and economic benefits for mothers and children [1, 5]. Despite such paramount importance, the South Asian region continues to grapple with achieving optimal early initiation of breastfeeding practice [6, 7]. A recent study indicated that the early initiation of breastfeeding rate in South Asia (< 45%) falls below the average early initiation of breastfeeding rate among lower middle-income countries (49.9%) [8]. Various individual, societal, geographical, and health-specific factors contribute to this low rate of early initiation of breastfeeding practice in South Asia, underscoring the necessity for maternal and child health interventions aimed at improving overall Infant and Young Child Feeding (IYCF) indicators, including early initiation of breastfeeding [9–12].

With a large population size and broadly similar healthcare structures, South Asia hosts the highest proportion of children living with malnutrition globally, largely attributed to suboptimal newborn care and IYCF practices, including early initiation of breastfeeding [13–16]. Therefore, global strategies for IYCF emphasize the need for comprehensive interventions to improve early initiation of breastfeeding practice [17]. Countries in South Asia, such as Bangladesh and India have implemented numerous national plans, policies and legislations to improve the IYCF components, including early initiation of breastfeeding [18, 19]. For example, the National Nutrition Program launched in 2004 in Bangladesh and IYCF Guidelines 2016 in India have highlighted the importance of improving early initiation of breastfeeding practice [20, 21]. These concerted efforts have resulted in a considerable change in the early initiation of breastfeeding rate in South Asia, increasing from 16% in 2000 to 40% in 2016 [14, 22], although it still remains below the desired level. One potential explanation for the failure to achieve the optimal level could be the presence of numerous interventions implemented within these policies, which exhibit varying degrees of effectiveness [23]. Some examples of interventions include educational and awareness programs targeting parents, community-based awareness initiatives, counseling programs, capacity building of the healthcare system, and interventions delivered through mobile phone-based technologies [23–25]. However, the magnitude of their overall effect and by intervention categories are still unknown. A systematic literature review and meta-analyses can fill the knowledge gap.

A previous scoping review identified 25 studies reporting early initiation of breastfeeding as an intervention outcome in South Asia [23]. That review included both experimental and quasi-experimental designs, had a liberal definition of early initiation of breastfeeding, e.g., breastfeeding initiation within three hours of birth, included studies published before 2018, and did not conduct a meta-analysis. Hence, the aim of this study is to systematically gather and analyze the evidence regarding the effectiveness of interventions on early initiation of breastfeeding in South Asian countries. Furthermore, the study aims to quantify the pooled effect of these interventions as well as their effectiveness by interventions categories.

## Methods

### Conceptualization

The Preferred Reporting Items for Systematic Review and Meta-Analysis (PRISMA) [26] was followed in this study. The protocol was registered in PROSPERO with ID: CRD42024462197. This review was based on a research plan that aimed to investigate the effects of interventions related to breastfeeding outcomes in South Asian countries. The breastfeeding outcomes encompassed several indicators, including early initiation of breastfeeding, colostrum feeding, exclusive breastfeeding, and continued breastfeeding. In this study, we specifically concentrate on early initiation of breastfeeding, which refers to the initiation of breastfeeding within one hour of birth.

### Data source and search strategy

A systematic literature search was conducted across five databases, including PubMed, Embase, Web of Science, CINAHL, and the Cochrane Library, using search strategies tailored to each database (Additional files, item 1). Given that this study involves a split review, the search strategy was developed considering all relevant breastfeeding indicators as outcome variables rather than being restricted solely to early initiation of breastfeeding.

### Inclusion and exclusion criteria

Inclusion and exclusion criteria were formulated in accordance with the PICOS framework (Table 1) [27]. Interventions implemented in South Asian countries, *i.e.* Afghanistan, Bangladesh, Bhutan, India, Maldives, Nepal, Pakistan, and Sri Lanka, where early initiation of breastfeeding was an outcome were included. The study population consisted of pregnant and lactating mothers without any clinical conditions. However, studies were also included where the intervention receivers were healthcare providers who ultimately delivered the interventions to pregnant and lactating mothers. Only randomized controlled trials (RCTs) were considered for inclusion, as RCTs provide the most robust evidence for causal relationships.

**Table 1:** Inclusion and exclusion criteria

Criteria	Inclusion criteria	Exclusion criteria
Population	Pregnant and lactating (PL) mothers in South Asian countries (Afghanistan, Bangladesh, Bhutan, India, Maldives, Nepal, Pakistan, and Sri Lanka)	Non-pregnant and non-lactating women, pregnant and lactating mothers living outside South Asian countries, PL mothers with any clinical condition
Intervention	Interventions targeting IYCF and maternal and child health which were delivered to PL mothers or health care providers	Interventions not targeting IYCF and infant and child health
Comparator	No intervention, usual care, alternative intervention, or minimal intervention	Nothing specified
Outcome	Early initiation of breastfeeding (breastfeeding within 1 hour of birth)	Other IYCF or essential newborn care components, e.g., exclusive breastfeeding, continuation of breastfeeding, complementary feeding, and thermal care
Study design	Randomized controlled trial (RCT) including cluster RCT	Observational and quasi-experimental studies
Others	Document type: peer-reviewed article; article language: English; publication period: any time before September 2023	Document type: unpublished data and grey literature; language: other than English

### Selection process

Following the literature search in the databases, records were retrieved and imported into EndNote for removing duplicates [28]. Thereafter, the records were imported into Covidence software for screening [29]. Selection of full texts was guided by inclusion and exclusion criteria. In every step, each record was screened by at least two reviewers, with conflicts resolved independently by a third reviewer.

### Data extraction and covariates

All included studies underwent narrative synthesis; therefore, summary tables were used to present background characteristics and intervention effects. For meta-analysis, we extracted information on proportion of mothers, in intervention and control groups, who practiced early initiation of breastfeeding and who did not. Several strategies were followed to include studies in the meta-analysis. For instance, in a study with multiple interventions and a single control group, the intervention groups were combined and compared with the control group. This was done to avoid duplication and unbalanced weight imparted by the identical control group [30, 31]. If the same effect size was reported in different studies published from the same intervention, only one of these was included. However, if the effects were measured at different follow-up points with data collected from different sets of participants, all such effect estimates were included in the meta-analysis.

### Data synthesis and meta-analysis

The meta-analysis approach was aimed to determine the overall relative risk (RR) of early initiation of breastfeeding among mothers in the intervention groups compared to those in the control groups [32, 33]. The meta-analysis was performed using the meta-analysis package of Stata software (version 17 StataCorp, College Station, Texas) [34]. A random effects model was used to estimate the pooled effect size considering the likelihood of intervention effects varying across designs, settings, and time [35]. A leave-one-out meta-analysis was conducted to estimate how the omission of any of the interventions effects the pooled estimate [36]. Meta funnel plot and Egger’s regression test were used to explore small study effects [37]. Subgroup meta-analysis was performed to observe the overall effect size by different groups including intervention types. Univariate and multivariate meta-regression were performed to explore the sources of heterogeneity upon observing a high degree of heterogeneity (I^2^ > 75%) [38]. Therefore, covariates such as country, settings, intervention type, number of comparison groups, intervention recipients, study design, study participants at baseline and endline, and the primary aim of interventions were taken into consideration. All the analyses were conducted considering *p* < 0.05 as statistical significance.

### Risk of bias and strength of evidence

The risk of bias of each study was assessed using version 2 of the Cochrane risk of bias assessment tools (RoB2) [39]. The risk of bias was evaluated across five domains: randomization, deviation from the intended intervention, missing outcome data, measurement of outcome, and selection of the reported results.

The strength of evidence was assessed by the Grading of Recommendations, Assessment, Development, and Evaluation (GRADE) criteria, considering domains related to risk of bias, inconsistency, indirectness, imprecision, publication bias, and overall effect [40]. The strength of evidence was assessed using the GRADEpro software [41].

## Results

### Selection process and study characteristics

**Fig. 1:**
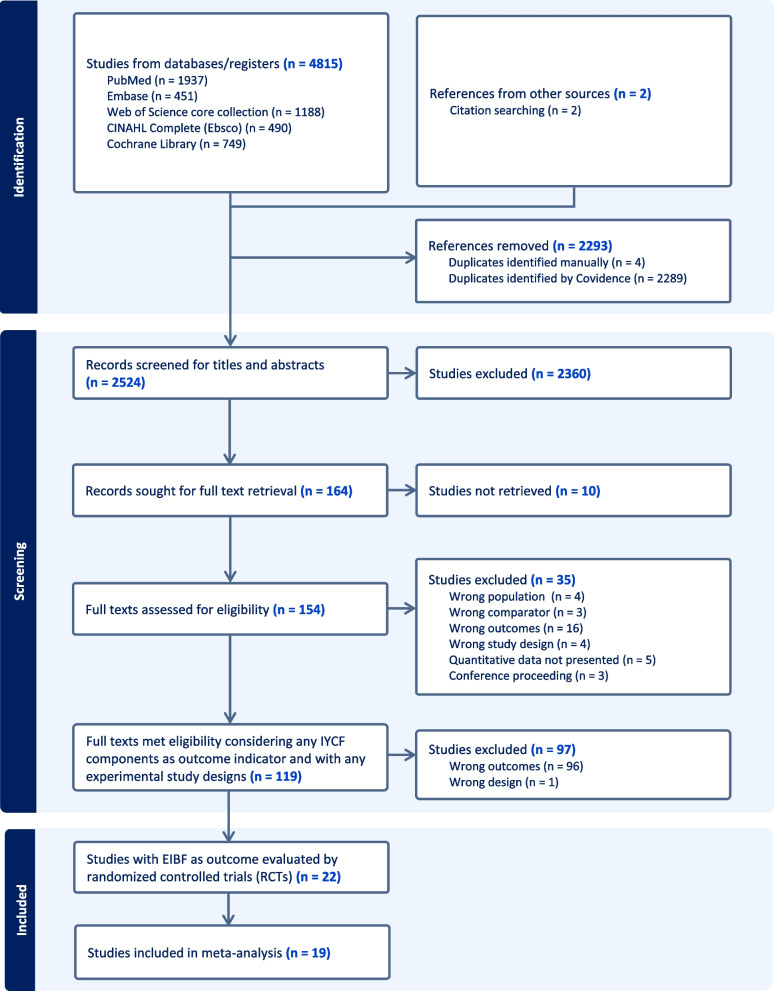
Selection of the included studies

A total of 4,817 records were retrieved from the considered databases and citation search. After removing duplicates, 2524 records were screened. After titles and abstracts and full texts screening, 22 studies were included in this review. Overall selection process is presented in Fig. 1. However, these 22 studies corresponded to 19 unique interventions because there were overlapping interventions for three pairs of included studies. For example, two studies reported the effect of same intervention at the same follow-up period [42, 43]. Similarly, another pair of studies had utilized data of the same intervention [44, 45]. Although the third pair of studies represented the same intervention, the effects were reported based on assessments considering different set of participants at different follow-up periods [46, 47].

The interventions were categorized based on their nature, recipients, and participants included in the baseline and endline assessments. Considering the nature, the interventions were broadly categorized into four types: behavioral, health system strengthening, mobile health (mHealth), and nutritional. We considered an intervention behavioral if it emphasized counseling and education session. An intervention was considered as health system strengthening if it focused on the capacity building of healthcare providers by providing training packages. Interventions employing the use of mobile phone-based technologies were labeled as mHealth. Interventions considering the provision of nutrient supplement were considered as nutritional. We also categorized interventions depending on whether they were primarily delivered to targeted mothers or healthcare providers. For some interventions, the baseline and endline participants were different. Therefore, the included interventions were also categorized based on whether the baseline and endline participants were the same or different. Background characteristics of the included studies, including country, settings, number of comparison groups, and baseline sample size, are summarized in Table 2.

**Table 2:** Background characteristics of included articles

Reference	Study	Country	Design	Setting	Number of arms	Baseline sample^α^
[42, 43]	Ara, 2018; Ara, 2019	Bangladesh	cRCT	Urban slum	2	Intervention:192 Control: 186
[48]	Bhutta, 2008	Pakistan	cRCT	Rural	2	Intervention: 2056 Control: 1691
[49]	Billah, 2022	Bangladesh	cRCT	Rural	5	Intervention: 1000 Control: 500
[50]	Carmichael, 2019	India	cRCT	Rural	2	Intervention: 769 Control: 790
[51]	Fottrell, 2013	Bangladesh	cRCT	Rural	2	Intervention:12,135 Control: 13,459
[52]	Gupta, 2019	India	RCT	Urban	2	Intervention: 150 Control: 150
[53]	Haider, 2000	Bangladesh	cRCT	Urban	2	Intervention:363 Control: 363
[54]	Jahan, 2014	Bangladesh	RCT	Urban	2	Intervention: 192 Control: 192
[46]	^β^Kim, 2018	Bangladesh	cRCT	Rural	2	Intervention:1095 Control: 1093
[55]	Kumar, 2008	India	cRCT	Rural	3	Intervention: 2749 Control: 1141
[56]	LeFevre, 2022	India	RCT	Urban and rural	2	Intervention: 2695 Control: 2400
[47]	^β^Menon, 2016	Bangladesh	cRCT	Rural	2	Intervention: 487 Control: 490
[57]	Modi, 2019	India	cRCT	Rural	2	Intervention: 4594 Control: 4740
[58]	Nguyen, 2017	Bangladesh	cRCT	Urban and rural	2	Intervention: 1300 Control: 1300
[59]	Nguyen, 2021	India	cRCT	Rural	2	Intervention:1237 Control: 1268
[60]	Saville, 2018	Nepal	cRCT	Rural	4	Intervention: 19,782 Control: 5310
[61]	Sikander, 2015	Pakistan	cRCT	Rural	2	Intervention: 224 Control: 228
[62]	Talukder, 2017	Bangladesh	cRCT	Rural	3	Intervention: 721 Control: 461
[44, 45]	Bhandari, 2012; Taneja, 2015	India	cRCT	Rural	2	Intervention:29,589 Control: 30,604
[63]	Ullah, 2019	Bangladesh	cRCT	Rural	4	Intervention: 1047 Control: 2964

^α^Number of samples were combined if there were more than one intervention groups; ^β^Kim, 2018 and Menon, 2016 represent the same intervention in Bangladesh although outcome was assessed at different time points with different groups of study participants; *RCT* Randomized Controlled Trial, *cRCT* Cluster Randomized Controlled Trial

Twelve of the 19 unique interventions fell into the behavioral category [43, 46, 49, 51–55, 58–61], three into the mHealth category [50, 56, 57], three into the health system strengthening category [45, 48, 62], and one into the nutritional supplement support category [63]. Healthcare professionals were the primary recipients of five interventions [45, 48, 50, 57, 62]. In six interventions, the primary outcome variable was not pertinent to breastfeeding but some other neonatal health-related indicators such as neonatal mortality, stillbirth, birth weight, and maternal dietary diversity [45, 48, 51, 55, 57, 60].

### Intervention effects and meta-analysis

Both positive and negative intervention effects were observed across the 19 interventions (Table 3) in the 22 studies. Among these, 15 interventions demonstrated a higher likelihood of early initiation of breastfeeding practices among mothers in intervention groups than that of mothers in control groups. One intervention demonstrated a null effect, indicating no significant difference in early initiation of breastfeeding practice between the intervention and control groups [63]. The remaining three interventions demonstrated negative effects [49, 56, 59], although the difference between intervention and control was not statistically significant in two interventions [49, 59] (Table 3).

**Table 3:** Intervention characteristics and their effects on early initiation of breastfeeding feeding (EIBF)

Reference	Inclusion criteria	Intervention component		Intervention duration	Program duration	Intervention effect
		**Intervention group**	**Comparison group**			
Ara, 2018 [42]; Ara, 2019 [43]	Pregnant women aged 16–49 years old, with no more than three living children or a parity of five and intended to reside in the area for at least 6 months after delivery	Peer IYCF counseling, psychosocial stimulation, feeding bowl and spoon, handwashing solution, and homemade toys	Usual care, feeding bowl and spoon	15 months (3rd trimester of pregnancy to 12 months of age of the child)	2 years	**EIBF:** 89.1% in intervention cluster and 77.4% in control cluster
Bhutta, 2008 [48]	Not specified	Training for lady health workers (LHWs) and *Dai* on basic newborn care Community organization and mobilization and group education sessions by LHWs and *Dai* trained in home-based basic newborn care	Usual LHW training program with regular refresher session	Three-monthly group education session attended by women of reproductive age, adolescent girls, and older	2 years	**EIBF**: 66.1% in intervention clusters and 21.1% in control clusters
Billah, 2022 [49]	Pregnant women within 125 days since the first date of the last menstrual period and permanent residents in the study village	**Group 1**: Nutrition specific behavior change communication (BCC) counseling, lipid-based prenatal nutrient supplement (PNS), lipid-based complementary nutrient supplement (CFS); **Group 2**: BCC + PNS; **Group 3**: BCC + CFS; **Group 4**: BCC	Usual care	Eight home-based counseling and practical demonstration (2 during pregnancy and 6 after delivery) from third trimester to six months after delivery	Not specified	Relative Risk (RR) of **EIBF** among intervention groups was 1.00 (95% CI: 0.93, 1.07, p = 0.929) compared to control
Carmichael, 2019 [50]	Women who had given birth in the catchment areas of the intervention and control sub-centers in the previous year	Information Communication Technology-Continuum of Care Services (ICT-CCS) program for health workers. ICT-CCS is a mobile phone-based job aid aimed to improve reproductive, maternal, newborn, child health and nutrition services (RMNCHN) and the *Ananya* program	*Ananya* program only	31 months	31 months	Difference in Difference (DiD) of EIBF between intervention and control was 14.7% (*p* < 0.01)
Fottrell, 2013 [51]	The study participants were women whose childbirth or death was recorded in the study areas	Participatory learning and action cycle in which women prioritize issues that affect maternal and neonatal health and design and implement strategies to address these issues. Health system strengthening initiatives from Diabetes Association of Bangladesh Perinatal Care Project	Health system strengthening initiatives from Diabetes Association of Bangladesh Perinatal Care Project	Monthly meeting by women’s groups continued for 30 months (from January 2009 to June 2011)	30 months	Compared to control, adjusted Odds Ratio of EIBF among intervention group was 1.16 (95% CI: 1.05, 1.28)
Gupta, 2019 [52]	≥ 18 years of age, gestational age of 18–22 weeks, singleton pregnancy, considering breastfeeding to newborn, planning to deliver in the same hospital, willing to stay in Aligarh for at least 6 months after delivery	Breastfeeding counseling (20–30 min long) by nutritionists trained in infant and young child feeding	Usual care	Two antenatal visits and eight postnatal visits from pregnancy to six months of childbirth	Not specified	EIBF was higher in the intervention group (73.4%) compared to the control group (33.6%) (*p* < 0.001)
Haider, 2000 [53]	Pregnant women aged 16–35 years, with no more than 3 living children or parity 5, who intended to stay in the area for at least 6 months after delivery	Peer counseling by local mothers trained in infant and young child feeding	No intervention	15 home-based counseling session from the last trimester to five months after delivery	Not specified	EIBF was 64% in intervention and 15% in control
Jahan, 2014 [54]	Women at a gestational age of 24 weeks attending the government Maternal and Child Health Training Institute	Nutrition education (increasing the food intake, preparation of nutrient-rich local food called Khichuri, breastfeeding, and maternal care) in the outpatient areas of the clinic to groups of six to eight women for one hour each month over 3-month period	Routine services from the health facility	One-hour nutrition education session on monthly basis from the third trimester of pregnancy	Three months	A statistically significant difference (*p* < 0.001) in EIBF practices between intervention (86%) and control groups (56.7%)
Kim, 2018 [46]	Households with children 0–5.9 months of age	Alive & Thrive program components: intensive interpersonal counseling (IPC), community mobilization (CM), and mass media (MM) at scale on IYCF	Standard care and less intensive nutrition counseling, CM and MM	48 months	48 months intervention and 24 months follow-up	At 6 years post baseline, EIBF was 70.5% in intervention and 54.8% in control, with adjusted DiD effect estimates 16.6. At 4 years post baseline, EIBF in intervention and control was respectively 94.3% and 75.7%, with an adjusted DiD 19.4
Kumar, 2008 [55]	Pregnant women	**Group 1**: A package of essential newborn care intervention; **Group 2**: Intervention package for group 1 plus liquid crystal hypothermia indicator	Usual care	From first trimester to post delivery, context specific intervention components were delivered	January 2004 to May 2005, total 16 months	Compared to control, Rate Ratios of EIBF among group 1 and group 2 were 4·57 (95% CI: 3·38–6·15, *p* < 0·0001) and 4·37 (95% CI: 3·23–5·90, *p* < 0·0001), respectively
LeFevre, 2022 [56]	Women with 12–34 weeks of gestation, more than 18 years of age, could speak and understand Hindi and owned or had access to a mobile phone	mHealth-based *Kilkari* message program consisting of prerecorded calls about reproductive, maternal, neonatal and child health (RMNCH) directly to families’ mobile phones	Usual care	Total 72 once weekly voice calls from the second trimester to 12 months post-delivery. 24 calls during pregnancy, 24 within the first 6 months postpartum, and 24 from 7 to 12 months postpartum	2018 to early 2020	Relative Risk of **EIBF** among intervention was 0.96 (95% CI: 0.92, 1.00, *p* = 0.027) compared to control
Menon, 2016 [47]	Pregnant women	Alive & Thrive program components: intensive interpersonal counseling (IPC), community mobilization (CM), and mass media (MM) at scale on IYCF	Standard care and less intensive nutrition counseling, CM and MM	48 months	48 months	At 4 years post baseline, EIBF in intervention and control was respectively 94.21% and 76.66%, with an adjusted DiD 16.70 (95% CI: 2.78, 30.57, *p* = 0.021)
Modi, 2019 [57]	All pregnant women, neonates, and infants from study area were the participants	Innovative Mobile-phone Technology for Community Health Operations (ImTeCHO), a mobile phone and web-based application, as a job aid to the government’s Accredited Social Health Activists (ASHAs) and Primary Health Center (PHC) staff to improve coverage of maternal, neonatal, and child health (MNCH)	Usual care	12 months (from February 2016 to January 2017)	12 months	Adjusted difference in mean of proportions by clusters regarding **EIBF** between intervention and control was 7.8 (95% CI: 4.2, 11.4) by intention-to-treat approach and was 6.7 (95% CI: 2.3, 11.2) by per protocol approach
Nguyen, 2017 [58]	Pregnant women and mothers with infants < 6 months of age	Nutrition-focused maternal, neonatal, and child health (MNCH) program on coverage of nutrition interventions, maternal dietary diversity, micronutrient supplement intake, and early breastfeeding practices	Standard MNCH (antenatal care with standard nutrition counseling)	16 months (from August 2015 to December 2016)	16 months	Statistically non-significant (p > = 0.05) adjusted DiD in **EIBF** between intervention (78.7%) and control (63.6%) was 12.7
Nguyen, 2021 [59]	Women with children < 6 months of age, pregnant women in the second and third trimesters	Nutrition-intensified antenatal care (I-ANC) component of Alive & Thrive program	Standard ANC in usual care	18 months (from June 2018 to December 2019)	18 months	Statistically non-significant difference (p > = 0.05) in EIBF between intervention (22%) and control (25%)
Saville, 2018 [60]	Married women aged 10–49 years, who had not had tubal ligation and whose husbands had not had vasectomy	**Group 1**: Participatory Learning and Action (PLA); **Group 2**: PLA plus food supplement; **Group 3**: PLA plus cash transfer	Usual care	14 months (29 Dec 2013 and 28 Feb 2015)	14 months	EIBF among group 1, group 2, group 3, and control was 38.1%, 39.6%, 52.5%, and 35.1%, respectively
Sikander, 2015 [61]	Pregnant women in third trimester	Cognitive-behavioral counseling by Lady Health Workers (LHW) to mothers	Routine counseling	7 cognitive behavioral counseling session from third trimester to six months postpartum	12 months (from May 2009 to April 2010)	Compared to control, adjusted Relative Risk of **EIBF** among intervention was 1.05 (95% CI: 0.85, 1.29, *p* = 0.65)
Talukder, 2017 [62]	Pregnant women in their second and/or third trimester) were identified as eligible for inclusion	Five-day training for the traditional birth attendants (TBAs)/community volunteers (CV) on breastfeeding **Group 1**: Breastfeeding promotion by trained TBAs/CV; **Group 2**: Breastfeeding promotion by trained TBAs/CV who were supervised	No intervention	Mothers in 2nd and 3rd trimester or with 0–6-month child were visited by TBA/CV for 6 months at irregular intervals	8 months	EIBF was higher among intervention groups (96%) than control group (88%)
Bhandari, 2012 [44]; Taneja, 2015 [45]	Not specified	Improving health workers skills on neonatal illness, strengthening the health system to implement Integrated Management of Neonatal and Childhood Illness (IMNCI)	Routine care	12 months (January to December 2007)	45 months (June 2006 to March 2010)	41% of the caregivers in the intervention clusters reported EIBF, compared with 11.2% in the control clusters (OR: 5.21, 95% CI: 4.33 to 6.28)
Ullah, 2019 [63]	Gestational age at 20 weeks, no plans to move away during pregnancy or the following 3 years	**Group 1**: Mothers and children received lipid-based nutrient supplement (LNS); **Group 2**: Mothers and children received iron and folic acid (IFA) and LNS, respectively; **Group 3**: Mothers and children received IFA and micronutrient powder (MNP), respectively	**Group 4**: Mothers received IFA and children received no supplement	Mothers received supplements from pregnancy to 6 months postpartum and children received supplements during 6–24 months of age	27–30 months	Rather than comparing intervention with control, LNS recipients and IFA recipients were compared. EIBF rate was 80.8% (*n* = 918) among mothers who received LNS and 81.4% (*n* = 2598) among mothers who received IFA

Note: *EIBF* Early Initiation of Breastfeeding, *IYCF* Infant and Young Child Feeding

Of the 22 studies, 19 were included in the meta-analysis. One study was excluded because intervention and control groups received two different interventions (lipid-based nutrient supplement vs. iron and folic acid supplement), representing more of a comparison between two interventions than the effect of a certain intervention [63]. The other two studies were excluded to avoid duplication bias [42, 44]. There were two studies representing the same interventions; however, these were included in the meta-analysis because the outcomes were assessed at different time points with different sets of samples [46, 47].

**Fig. 2:**
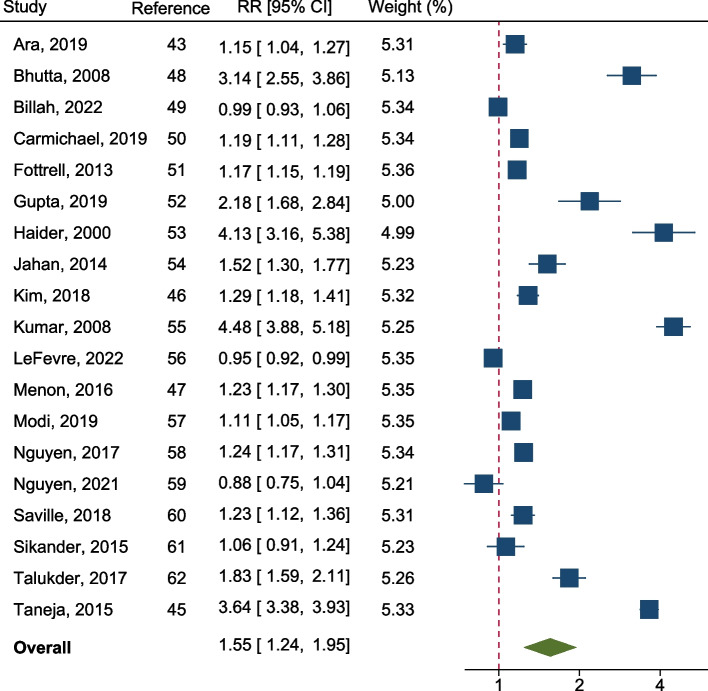
Overall Relative Risk (RR) of early initiation of breastfeeding among mothers who received interventions compared to their counterparts

The pooled RR of practicing early initiation of breastfeeding among mothers in the intervention groups compared to that of mothers in the control groups was 1.55 (95% CI: 1.24, 1.95; *p* < 0.001), indicating intervention receiving mothers had a 55% higher likelihood of practicing early initiation of breastfeeding than their counterparts. The degree of heterogeneity (I^2^) across the intervention effects was 99.56% (Fig. 2). Sensitivity analysis, as conducted by leave-one-out meta-analysis, identified that omission of any interventions did not result in significant changes in the pooled intervention effect, indicating a high stability of the overall effect (Additional files, item 2). The meta-funnel plot showed that the studies were evenly spread out on both sides of the mean. However, there were too many large studies in the upper part of the funnel, which suggests that there was a small study effect or publication bias (Egger's *p* = 0.003) (Additional files, item 3). Study specific characteristics of the covariates are presented in the Additional files, item 4. Subgroup analysis revealed a significant difference in the effect size between interventions categorized by settings (rural, urban, and both) and intervention types (behavioral, system strengthening, and mHealth) (Additional files, item 5). Concerning the types of interventions, the pooled RR of early initiation of breastfeeding practices among mothers who were in behavioral, health system strengthening, and mHealth, as compared to controls, were 1.48 (95% CI: 1.14, 1.93), 2.76 (95% CI: 1.96, 3.88), and 1.08 (95% CI: 0.97, 1.20), respectively. In meta-regression, type of intervention (*p* < 0.001) and primary outcome (*p* = 0.017) variables demonstrated a significant association with the overall intervention effect. The certainty (R^2^) and significance level (p) of the adjusted meta-regression model were 43.92% and < 0.001, respectively (Additional files, item 6).

### Risk of bias and strength of evidence

Of the 22 articles, six of these articles raised some concerns, while the remaining 16 showed a high risk of bias (Additional files, item 7). Issues concerning the risk of bias were more frequently observed in the domains of randomization, deviation from intended interventions, and selection of reported results. Regarding randomization, 12 studies were assessed as having some concerns, and four studies had a high risk of bias. In the domains of deviation from intervention and selection of reported results, six studies each were found to have some concerns regarding the risk of bias (Additional files, item 7). The overall strength of evidence of meta-analysis was found to be moderate (Additional files, item 8).

## Discussion

The meta-analysis revealed that mothers in the intervention groups had a 1.55 times higher likelihood of practicing early initiation of breastfeeding than mothers in the control groups. Although the overall strength of evidence was moderate and bias was observed in individual studies, the pooled effect still considerably favors the interventions in improving early initiation of breastfeeding coverage in South Asia. Interventions targeting health system strengthening were more effective than behavioral and mHealth interventions.

Among the considered interventions, only three exhibited negative effects (RR < 1) on early initiation of breastfeeding [49, 56, 59]. Of these, one intervention delivered IYCF messages to the mobile phones of the participants in the intervention group. However, reports suggest that a one-way communication channel and potential conflict with prevalent traditional practices in the community may have resulted in a poor translation of educational messages into practice [56]. The other two studies highlighted that there was a consistent change in early initiation of breastfeeding practice over time in Bangladesh, regardless of the implementation of any specific interventions, causing the intervention effect to fade out [49, 59]. Considering the post-delivery physiological and psychological condition of mothers, support from healthcare providers during and immediately after delivery emerges as crucial to ensure early initiation of breastfeeding [47, 59]. This might explain why interventions delivered to healthcare providers exhibited a stronger effect than those delivered to mothers (Additional files, item 5).

The meta-analysis demonstrated a high degree of heterogeneity (I^2^ = 99.56). The degree of heterogeneity remained high even when the interventions were categorized by types, country, settings, comparison groups, intervention recipients, samples at baseline and endline, study designs, and primary outcomes. Meta-regression identified intervention type and recipient together explained the uncertainty (R^2^) level by 43.92% (*p* < 0.001). There might be other possible sources of heterogeneity, e.g., variation in intervention components, variation in mode of intervention delivery, and variation in the population and sociodemographic characteristics, which we were not able to explore. Interventions that did not primarily target breastfeeding were associated with a 53% higher likelihood of early initiation of breastfeeding among mothers, compared to those interventions that specifically targeted breastfeeding indicators (Additional files, item 6). Data showed that interventions primarily did not target breastfeeding but essential newborn care were mainly health system strengthening in nature and were delivered to healthcare providers, compared to those targeting breastfeeding indicators only (Additional files, item 6). This aligns with the idea that healthcare providers trained in newborn care are better equipped to support mothers in practicing early initiation of breastfeeding after delivery.

No relevant intervention was found in four South Asian countries, including Afghanistan, Bhutan, Maldives, and Sri Lanka, indicating a disproportionate allocation of interventions, especially considering the need in country like Afghanistan where early initiation of breastfeeding practice remains lower than others [22].

The assessment of the risk of bias in the included studies revealed several critical factors that may have contributed to the observed biases across different domains. In the outcome measures domain, studies that demonstrated a low risk of bias often involved outcome assessors or field investigators who were different from the intervention providers. This separation of roles likely contributed to a more objective assessment process, reducing potential assessment bias. Moreover, studies with a low risk of bias in this domain frequently collected data within 72 hours of delivery, which minimized the chances of recall bias and reporting bias, as the outcomes were recorded while memories were still recent and accurate.

Some RCTs did not provide enough information on the randomisation domain especially on randomization concealment (Additional files, item 7). Thus, these studies were assessed having a high risk of bias in this domain. The inherent difficulty in concealing interventions from participants and providers, given the nature of the interventions, likely exacerbated this issue. Without proper concealment, there is a greater risk of selection bias, where the knowledge of group assignments could influence the allocation process and outcomes. However, concealment of allocation is highly challenging for community-based interventions in real-world settings [64]. The deviation from intervention domain also showed a high risk of bias, which can be attributed to the lack of clear reporting on whether the interventions were delivered as intended and the adherence of participants to the intervention protocols. Some studies did not adequately document whether the interventions were delivered as planned or if participants adhered to the prescribed interventions (Additional files, item 7). Moreover, the nature of the interventions, such as counseling sessions for mothers, made it difficult to conceal them from the providers. This awareness could raise the risk of reporting bias. The strength of evidence was found to be moderate, which is mainly because of serious bias, serious inconsistency, and the presence of small study effects. While the overall effect consistently favors the intervention, individual interventions demonstrated both positive and negative outcomes, leading to inconsistencies. Furthermore, the presence of a considerably large effect, especially for outcomes like early initiation of breastfeeding, also impacted the overall strength of the evidence.

Several strengths of this study are noteworthy. Rigorous literature searches across the most relevant databases without publication time limitations likely facilitated the identification of a higher number of relevant records. The inclusion of studies with randomized experimental designs added more strength to the synthesized evidence. The degree of heterogeneity was further explored using meta-regression. Apart from strengths, certain limitations also warrant mentioning. Firstly, interventions which are complex in nature are challenging to tag under a certain category. For example, a mobile phone-based intervention may encompass both behavioral and health system strengthening aspects [56, 57]. Secondly, studies with a long follow-up period had different sets of samples at baseline and endline, rendering domains related to “missingness” inapplicable. Thirdly, for meta-analysis, the number of successes and failures in intervention and control groups was extracted instead of considering the effect estimates reported in individual studies. This could potentially result in a different pooled effect size had the reported effect sizes in individual studies been considered. Fourthly, despite the commendable success of the interventions indicated by the pooled effect size, the moderate strength of evidence and presence of bias in individual studies should be considered while extrapolating the results. Finally, the current data indicates that some South Asian countries have not implemented any interventions. This does not indicate the absence of policies and programs targeting early initiation of breastfeeding in these countries, but highlights that these policies and programs were probably not evaluated by RCT [65, 66]. Therefore, these issues can be taken into careful consideration while extrapolating the findings to South Asia.

This study holds policy importance because it summarizes the interventions demonstrating an effect on early initiation of breastfeeding practices in South Asia. Given the sociocultural similarities in maternal and newborn care in this region, the best practices identified can be considered for further contextualization and implementation in the areas with low early initiation of breastfeeding practice. Successful and feasible strategies can be integrated into breastfeeding related programs and policies. There is scope to understand the equity aspects and sustainability of these interventions, which could be a focus of future research.

## Conclusion

The interventions implemented in South Asian countries appeared to be successful in enhancing the early initiation of breastfeeding practice among mothers. Interventions focused on strengthening health systems proved to be more effective than other approaches, highlighting the role of health system improvements in promoting breastfeeding outcome. Findings can serve as a valuable resource to formulate effective and feasible interventions targeting communities experiencing persistently low early initiation of breastfeeding rate in this region, taking the moderate strength of evidence and risk of bias into careful consideration.

### Ethical considerations

In this study, published peer-reviewed research articles served as source of data, obviating the need for obtaining informed consent and ethical approval. However, the presence of publication bias remains as an ethical concern because it holds the potential to mislead aggregated effect estimates. Despite this inherent risk, this study systematically summarized all pertinent evidence concerning the effect of maternal and child health interventions on early initiation of breastfeeding practice in South Asia. The implication of our findings may extend to policymaking realms. Furthermore, use of validated tools such as PRISMA as reporting guidelines underscore the robustness of approach. Therefore, considering the methodological rigor and the public health significance of the topic, the potential benefits might outweigh the anticipated risks.

## Supplementary Information

Supplementary Material 1.

## Data Availability

No datasets were generated or analysed during the current study.
