# Supplementary material for: Effectiveness of interventions on early initiation of breastfeeding in South Asia: a systematic review and meta-analysis of randomized controlled trials
Source: Int Breastfeed J. 2025 May 27;20:43. doi: 10.1186/s13006-025-00736-2 (PMC12117840; doi:10.1186/s13006-025-00736-2)
Supplement: Supplementary file 1 — Supplementary Material 1. [file 13006_2025_736_MOESM1_ESM.pdf]

Effectiveness of maternal and child health interventions on early initiation of breastfeeding in South Asia: A systematic review and meta-analysis

Supplementary file

Table of Contents

Items Page number

1 Search strategy 2
2 Leave-one-out meta-analysis 6
3 Publication bias 7
3.1 Meta Funnel plot 7
3.2. Egger's regression test 7
4 Characteristics of included studies 8
5 Subgroup analysis 9
6 Meta-regression analysis 10
7 Assessment of risk of bias 11
8 Strength of evidence 12
9 Funding sources 13
10 PRISMA checklist 14

1 Search strategy

Table S1: Search strategy

Database
**PubMed (National Library of Medicine)**<br>Date of search: 20230911<br>#1<br>"Asia, Southern"[Mesh]<br>=174847<br><br>#2<br>bangladesh OR bangladeshi OR india OR indian OR indians OR pakistan OR pakistani OR "sri lanka" OR "sri lankan" OR "sri lankans" OR ceylon OR nepal OR nepalese OR bhutan OR bhutanese OR maldives OR maldivian OR maldivians OR afghanistan OR afghani OR afghan OR afghans OR "south asia" OR "south asian" OR "south asians" OR "southern asia" OR "southern asian" OR "southern asians"<br>=1098749
#3<br>#1 OR #2<br>=1098749<br><br>#4<br>(((("Breast Feeding"[Mesh]) OR "Colostrum"[Mesh]) OR "Breast Milk Expression"[Mesh]) OR "Lactation"[Mesh]) OR "Bottle Feeding"[Mesh]<br>=93923<br><br>#5<br>breastfeed* OR "breast feed*" OR "breast milk expression*" OR colostrum* OR "breast milk expression*" OR "feeding breast*" OR lactat* OR breastfed OR "breast fed" OR "prelacteal feed*" OR "bottle feed*" OR "formula feed*" OR "breastmilk substitute*" OR "breast milk substitute*"<br>=314801<br><br>#6<br>#4 OR #5<br>=314902<br><br>#7<br>"Randomized Controlled Trial" [Publication Type]<br>=600968<br><br>#8<br>intervent* OR experiment* OR randomised OR randomized OR "quasi-experimental" OR "quasi experimental" OR quasiexperimental OR RCT<br>=5784785<br><br>#9<br>#7 OR #8<br>=5785186<br><br>#10<br>#3 AND #6 AND #9<br>=2758

#11
#10 Filters: Other Animals
=823

#12
#10 NOT #11
=**1937 records**

Embase (Elsevier)
Date of search: 20230911
#1
'south asia'/exp OR 'south asian'/exp OR bangladesh:ti,ab,kw OR bangladeshi:ti,ab,kw OR india:ti,ab,kw OR indian:ti,ab,kw OR indians:ti,ab,kw OR pakistan:ti,ab,kw OR pakistani:ti,ab,kw OR 'sri lanka':ti,ab,kw OR 'sri lankan':ti,ab,kw OR 'sri lankans':ti,ab,kw OR ceylon:ti,ab,kw OR nepal:ti,ab,kw OR nepalese:ti,ab,kw OR bhutan:ti,ab,kw OR bhutanese:ti,ab,kw OR maldives:ti,ab,kw OR maldivian:ti,ab,kw OR maldivians:ti,ab,kw OR afghanistan:ti,ab,kw OR afghani:ti,ab,kw OR afghan:ti,ab,kw OR afghans:ti,ab,kw OR 'south asia':ti,ab,kw OR 'south asian':ti,ab,kw OR 'south asians':ti,ab,kw OR 'southern asia':ti,ab,kw OR 'southern asian':ti,ab,kw OR 'southern asians':ti,ab,kw
=458233

#2
'breast feeding'/exp OR 'breast milk expression'/exp OR 'colostrum'/exp OR 'lactation'/exp OR 'bottle feeding'/exp OR breastfeed*:ti,ab,kw OR 'breast feed*':ti,ab,kw OR colostrum*:ti,ab,kw OR 'breast milk expression*':ti,ab,kw OR 'feeding breast*':ti,ab,kw OR lactat*:ti,ab,kw OR breastfed:ti,ab,kw OR 'breast fed':ti,ab,kw OR 'prelacteal feed*':ti,ab,kw OR 'bottle feed*':ti,ab,kw OR 'formula feed*':ti,ab,kw OR 'breastmilk substitute*':ti,ab,kw OR 'breast milk substitute*':ti,ab,kw
=340581

#3
'randomized controlled trial'/exp OR intervent*:ti,ab,kw OR experiment*:ti,ab,kw OR randomised:ti,ab,kw OR randomized:ti,ab,kw OR 'quasi-experimental':ti,ab,kw OR 'quasi experimental':ti,ab,kw OR quasiexperimental:ti,ab,kw OR rct:ti,ab,kw
=5611075

#4
#1 AND #2 AND #3
=1461

#5
#4 AND ('animal experiment'/de OR 'animal model'/de OR 'animal tissue'/de OR 'nonhuman'/de)
=149

#6
#4 NOT #5
=1312

#7
#6 AND [embase]/lim NOT ([embase]/lim AND [medline]/lim)
=**451 references**

Web of Science core collection (Clarivate)
Date of search: 20230912
#1
TS=(bangladesh OR bangladeshi OR india OR indian OR indians OR pakistan OR pakistani OR "sri lanka" OR "sri

lankan" OR "sri lankans" OR ceylon OR nepal OR nepalese OR bhutan OR bhutanese OR maldives OR maldivian OR maldivians OR afghanistan OR afghani OR afghan OR afghans OR "south asia" OR "south asian" OR "south asians" OR "southern asia" OR "southern asian" OR "southern asians")
=714702

#2
TS=(breastfeed* OR "breast feed*" OR "breast milk expression*" OR colostrum* OR "breast milk expression*" OR "feeding breast*" OR lactat* OR breastfed OR "breast fed" OR "prelacteal feed*" OR "bottle feed*" OR "formula feed*" OR "breastmilk substitute*" OR "breast milk substitute*")
=280036

#3
TS=(intervent* OR experiment* OR randomised OR randomized OR "quasi-experimental" OR "quasi experimental" OR quasiexperimental OR RCT)
=9254807

#4
#1 AND #2 AND #3
=**1188 records**

CINAHL Complete (Ebsco)
Date of search: 20230912
#1
(MH "Asia, Southern+") OR (bangladesh OR bangladeshi OR india OR indian OR indians OR pakistan OR pakistani OR "sri lanka" OR "sri lankan" OR "sri lankans" OR ceylon OR nepal OR nepalese OR bhutan OR bhutanese OR maldives OR maldivian OR maldivians OR afghanistan OR afghani OR afghan OR afghans OR "south asia" OR "south asian" OR "south asians" OR "southern asia" OR "southern asian" OR "southern asians"))
=97424

#2
((MH "Breast Feeding+") OR (MM "Colostrum") OR (MM "Lactation") OR (MM "Bottle Feeding")) OR (breastfeed* OR "breast feed*" OR "breast milk expression*" OR colostrum* OR "breast milk expression*" OR "feeding breast*" OR lactat* OR breastfed OR "breast fed" OR "prelacteal feed*" OR "bottle feed*" OR "formula feed*" OR "breastmilk substitute*" OR "breast milk substitute*")
=62030

#3
(MH "Randomized Controlled Trials+") OR TI (intervent* OR experiment* OR randomised OR randomized OR "quasi-experimental" OR "quasi experimental" OR quasiexperimental OR RCT) OR AB (intervent* OR experiment* OR randomised OR randomized OR "quasi-experimental" OR "quasi experimental" OR quasiexperimental OR RCT)
=939838
#4
#1 AND #2 AND #3
=**490 records**

Cochrane library (Cochrane central)
Date of search: 20230912
#1
MeSH descriptor: [Asia, Southern] explode all trees
=5980

#2
(bangladesh OR bangladeshi OR india OR indian OR indians OR pakistan OR pakistani OR "sri lanka" OR "sri lankan" OR "sri lankans" OR ceylon OR nepal OR nepalese OR bhutan OR bhutanese OR maldives OR maldivian

OR maldivians OR afghanistan OR afghani OR afghan OR afghans OR "south asia" OR "south asian" OR "south asians" OR "southern asia" OR "southern asian" OR "southern asians"):ti,ab,kw
=21612

#3
#1 OR #2
=21612

#4
MeSH descriptor: [Breast Feeding] explode all trees
=2674

#5
MeSH descriptor: [Colostrum] explode all trees
=216

#6
MeSH descriptor: [Lactates] in all MeSH products
=3565

#7
MeSH descriptor: [Bottle Feeding] explode all trees
=252

#8
(breastfeeding OR "breast feeding" OR breastfeed OR "breast feed""breast milk expression" OR "breast milk expressions" OR colostrum OR "feeding breast" OR lactating OR lactation OR lactates OR breastfed OR "breast fed" OR "prelacteal feeding" OR "bottle feeding" OR "formula feeding" OR "breastmilk substitute" OR "breastmilk substitutes" OR "breast milk substitute" OR "breast milk substitutes"):ti,ab,kw
=28975

#9
#4 OR #5 OR #6 ORR #7 OR #8
=29657

#10
(intervent* OR experiment* OR randomised OR randomized OR "quasi-experimental" OR "quasi experimental" OR quasiexperimental):ti,ab,kw
=1501061

#11
#3 AND #9 AND #10
=749 records trials 731

2 Leave-one-out meta-analysis

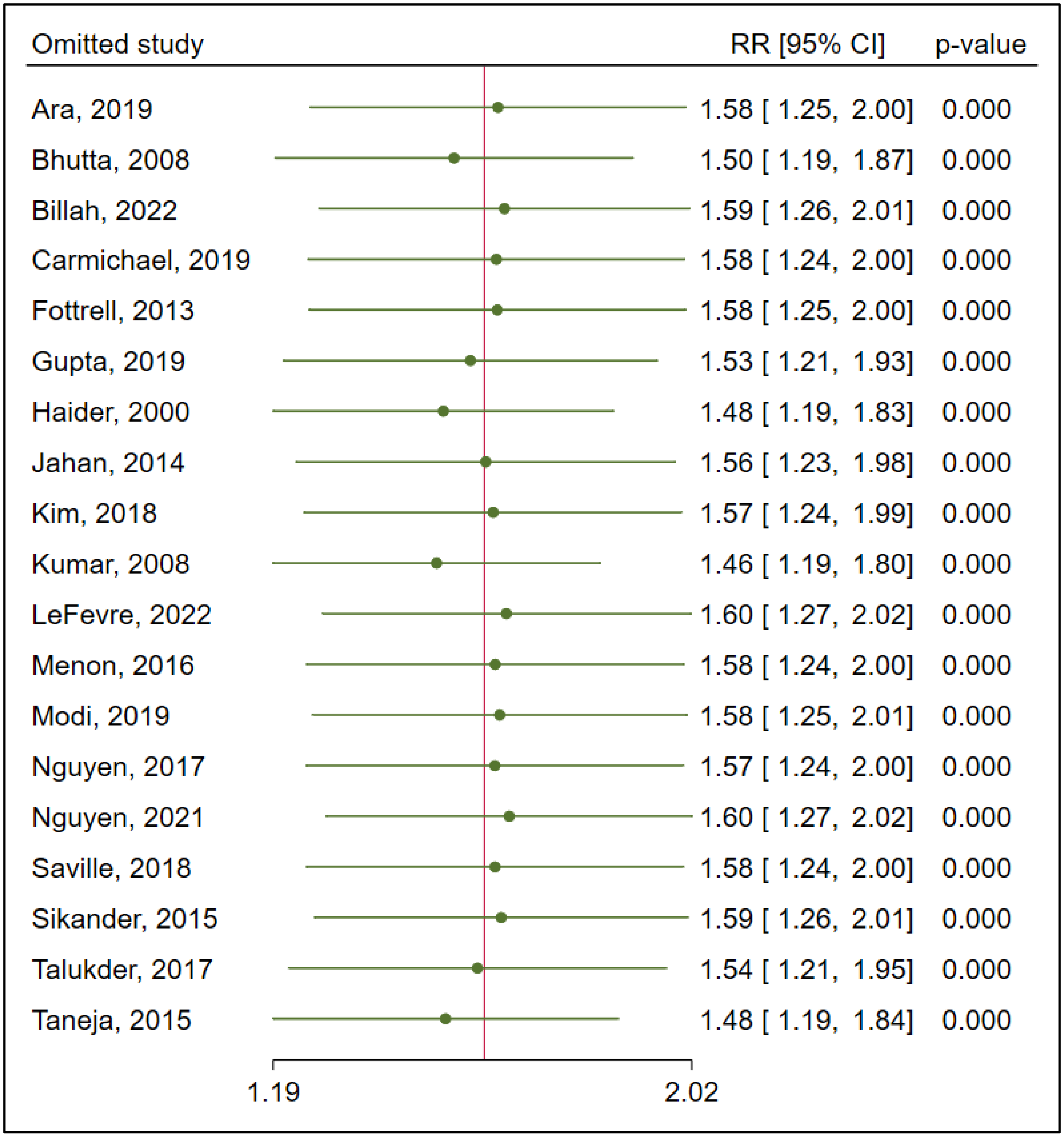

Figure 1 Leave-one-out meta-analysis

3 Publication bias

3.1 Meta Funnel plot

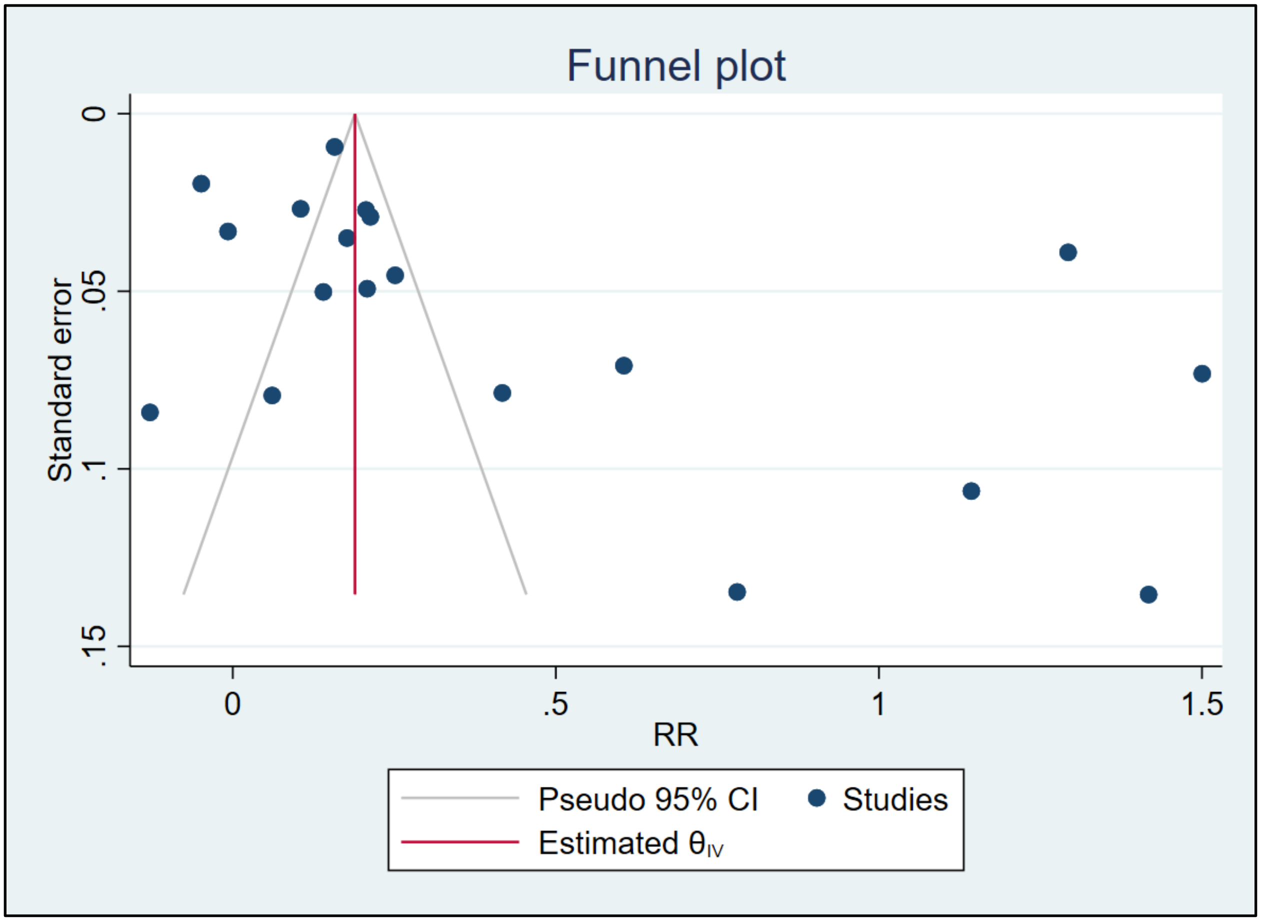

Figure 2 Meta Funnel plot

3.2. Egger's regression test

H0: beta1 = 0; no small-study effects
beta1 = 7.91
SE of beta1 = 2.713
z = 2.91
Prob > |z| = 0.0036

4 Characteristics of included studies

Table S2: characteristic of included studies by country, settings, intervention type, study arms, intervention recipients, baseline and endline participants, designs, and primary outcomes.

Author	Country	Settings	Intervention type	Study arms	Intervention recipient	Baseline and endline participants	Design	Primary outcome
Ara, 2019	Bangladesh	Urban	Behavioral	2	Mothers	Same	cRCT	Breastfeeding
Bhutta, 2008	Pakistan	Rural	System strengthening	2	Healthcare providers	Different	cRCT	Not breastfeeding
Billah, 2022	Bangladesh	Rural	Behavioral	> 2	Mothers	Same	cRCT	Breastfeeding
Carmichael, 2019	India	Rural	mHealth	2	Healthcare providers	Different	cRCT	Breastfeeding
Fottrell, 2013	Bangladesh	Rural	Behavioral	2	Mothers	Different	cRCT	Not breastfeeding
Gupta, 2019	India	Urban	Behavioral	2	Mothers	Same	RCT	Breastfeeding
Haider, 2000	Bangladesh	Urban	Behavioral	2	Mothers	Same	cRCT	Breastfeeding
Jahan, 2014	Bangladesh	Urban	Behavioral	2	Mothers	Same	RCT	Breastfeeding
Kim, 2018	Bangladesh	Rural	Behavioral	2	Mothers	Different	cRCT	Breastfeeding
Kumar, 2008	India	Rural	Behavioral	> 2	Mothers	Same	cRCT	Not breastfeeding
LeFevre, 2022	India	Both	mHealth	2	Mothers	Same	RCT	Breastfeeding
Menon, 2016	Bangladesh	Rural	Behavioral	2	Mothers	Different	cRCT	Breastfeeding
Modi, 2019	India	Rural	mHealth	2	Healthcare providers	Different	cRCT	Not breastfeeding
Nguyen, 2017	Bangladesh	Both	Behavioral	2	Mothers	Different	cRCT	Breastfeeding
Nguyen, 2021	India	Rural	Behavioral	2	Mothers	Different	cRCT	Breastfeeding
Saville, 2018	Nepal	Rural	Behavioral	> 2	Mothers	Same	cRCT	Not breastfeeding
Sikander, 2015	Pakistan	Rural	Behavioral	2	Mothers	Same	cRCT	Breastfeeding
Talukder, 2017	Bangladesh	Rural	System strengthening	> 2	Healthcare providers	Different	cRCT	Breastfeeding
Taneja, 2015	India	Rural	System strengthening	2	Healthcare providers	Different	cRCT	Not breastfeeding
Ullah, 2019	Bangladesh	Rural	Nutritional	> 2	Mothers	Same	cRCT	Breastfeeding

5 Subgroup analysis

Table S3: Intervention effects by subgroups

Subgroups	Number of interventions	RR (95% CI)	I^2(%)	p-heterogeneity	p-Egger	p-difference
**Country**						
Bangladesh	9	1.44 (1.23, 1.84)	99.26	< 0.001	< 0.001	0.291
India	7	1.69 (1.07, 2.70)	99.62	< 0.001		
Nepal	1	1.23 (1.12, 1.36)	-	-		
Pakistan	2	1.82 (0.86, 3.85)	97.00	< 0.001		
**Settings**						
Rural	13	1.53 (1.16, 2.02)	99.57	< 0.001	< 0.001	**0.018**
Urban	4	1.97 (1.23, 3.13)	96.51	< 0.001		
Both	2	1.08 (0.90, 1.30)	96.41	< 0.001		
**Intervention type**						
Behavioral	13	1.48 (1.14, 1.93)	99.43	< 0.001	< 0.001	**< 0.001**
System strengthening	3	2.76 (1.96, 3.88)	94.85	< 0.001		
mHealth	3	1.08 (0.97, 1.20)	92.15	< 0.001		
**Comparison groups**						
Two	15	1.50 (1.18, 1.91)	99.55	< 0.001	< 0.001	0.589
More than two	4	1.76 (1.01, 3.12)	99.16	< 0.001		
**Intervention recipient**						
Mothers	14	1.44 (1.12, 1,85)	99.54	< 0.001	< 0.001	0.238
Healthcare providers	5	1.94 (1.26, 2.97)	99.22	< 0.001		
**Baseline and endline sample**						
Different	10	1.49 (1.14, 1.96)	99.53	< 0.001	< 0.001	0.701
Same	9	1.63 (1.13, 2.36)	99.23	< 0.001		
**Study design**						
cRCT	16	1.58 (1.22, 2.04)	99.58	< 0.001	< 0.001	0.697
RCT	3	1.44 (0.98, 2.11)	95.59	< 0.001		
**Primary outcome**						
Breastfeeding	13	1.35 (1.10, 1.67)	98.80	< 0.001	< 0.001	0.102
Not related breastfeeding	6	2.08 (1.30, 3.33)	99.69	< 0.001		

6 Meta-regression analysis

Table S4: Meta-regression analysis

Covariates	Univariable model			Adjusted model[α]	
	RR (95% CI)	p-value	R^2	ARR	p-value
**Country**		**0.679**	**0.00**		
Bangladesh	Reference	-			
India	1.17 (0.82, 1.67)	0.389			
Nepal	0.86 (0.41, 1.8)	0.682			
Pakistan	1.26 (0.72, 2.2)	0.423			
**Settings**		**0.140**	**0.00**		
Both	Reference	-			
Rural	1.41 (0.85, 2.32)	0.181			
Urban	1.79 (1, 3.19)	0.049			
**Intervention type**		**0.000**	**49.06**		
Behavioral	Reference	-		Reference	-
System strengthening	1.9 (1.42, 2.55)	0.000		1.57 (0.83, 2.97)	0.164
mHealth	0.74 (0.56, 0.99)	0.040		0.65 (0.41, 1.05)	0.080
**Comparison groups**		**0.348**	**0.00**		
Two	Reference	-			
More than two	1.19 (0.83, 1.7)	0.348			
**Intervention recipient**		**0.062**	**2.73**		
Healthcare providers	Reference	-		Reference	-
Mothers	0.74 (0.54, 1.02)	0.062		0.83 (0.47, 1.45)	0.506
**Baseline and endline sample**		**0.614**	**0.00**		
Different	Reference	-			
Same	1.08 (0.8, 1.47)	0.614			
**Study design**		**0.691**	**0.00**		
RCT	Reference	-			
cRCT	1.09 (0.72, 1.65)	0.691			
**Primary outcome**		**0.017**	**0.00**		
Breastfeeding	Reference	-			
Not breastfeeding	1.53 (1.08, 2.16)	0.017			

[α]Adjusted model (R^2=43.92, p<0.001)

7 Assessment of risk of bias

Table S5: Risk of bias for studies with cluster randomized design

Study	Randomization	Recruitment	Deviation from intervention	Missing outcome	Outcome measure	Selection of reported result	Overall
Ara, 2018<br>Ara, 2019	High	Low	Low	Low	Some concerns	Low	High
Bhutta, 2008	Some concerns	Low	Low	Low	Some concerns	Some concerns	High
Billah, 2022	Low	Some concerns	Some concerns	Low	Some concerns	Low	High
Carmichael, 2019	High	Low	Low	Low	Low	Low	High
Fottrell, 2013	Low	Low	Some concerns	Low	Low	Low	Some concerns
Haider, 2000	Low	Low	Low	Some concerns	Some concerns	Some concerns	High
Kim, 2018	Some concerns	Low	Low	Low	Low	Some concerns	High
Kumar, 2008	Low	Low	Some concerns	Low	Low	Some concerns	High
Menon, 2016	Some concerns	Low	Some concerns	Low	Low	Some concerns	High
Modi, 2019	Some concerns	Low	Low	Low	Low	Low	Some concerns
Nguyen, 2017	Some concerns	Low	Some concerns	Low	Some concerns	Low	High
Nguyen, 2021	Some concerns	Low	Low	Low	Low	Some concerns	High
Saville, 2018	High	Low	Low	Low	High	Low	High
Sikandar, 2015	Some concerns	Some concerns	Some concerns	Low	Low	Low	High
Talukder, 2017	Low	Some concerns	Low	Low	Low	Low	Some concerns
Taneja, 2015<br>Bhandari, 2012	Some concerns	Some concerns	Low	Low	Low	Low	High
Ullah, 2019	Some concerns	Low	Low	Low	Low	Low	Some concerns

Table S6: Risk of bias for studies with individually randomized RCT

Study	Randomization	Deviation from intervention	Missing outcome	Outcome measure	Selection of reported result	Overall
Gupta, 2019	Some concerns	Low	Low	Low	Low	Some concerns
Jahan, 2014	Some concerns	Low	Low	Low	Low	Some concerns
LeFevre, 2022	High	Some concerns	Low	Low	Low	High

8 Strength of evidence

Table S7: Strength of evidence from the meta-analysis according to GRADE criteria

**Certainty assessment**										**Summary of findings**				**Certainty level (high, moderate, low, very low)**
No. of studies	Design (Randomized trial, observational)	Bias (Not serious, serious, very serious)	Inconsistency (Not serious, serious, very serious)	Indirectness (Not serious, serious, very serious)	Imprecision (Not serious, serious, very serious)	Publication bias (Undetected, strongly suspected)	Large effect (No, large, very large)	Confounding (No, would reduce demonstrated effect, would suggest spurious effect)	Does response gradient (No, yes)	**Number of participants**		**Effect**		
										Intervention	Comparison	Relative (95% CI)	Absolute (95% CI)	
19	Randomized trial	Serious	Serious	Not serious	Not serious	Strongly suspected	Very large	No	No	19412/30484 (63.7%)	12399/26319 (47.1%)	RR 1.55 (1.24, 1.95)	259 more per 1000 (from 113 more to 448 more)<br>0 fewer per 1000 (from 0 fewer to 0 fewer)	**Moderate**

Consistency: Small variation in effects, CIs overlap, low heterogeneity (I^2)
Directness: Generalizability, transferability, applicability, external validity
Precision: High sample size, high frequency of outcome, narrow confidence interval, low standard error

9 Funding sources

Table S8: Finding sources

Reference	Country	Funding sources
Ara, 2018; Ara, 2019	Bangladesh	Stars in Global Health, Grand Challenges Canada
Bhutta, 2008	Pakistan	The Hala project is supported by a collaborative grant from WHO and the Saving Newborn Lives (SNL) program of Save the Children (USA), funded by the Bill & Melinda Gates Foundation
Billah, 2022	Bangladesh	This study was funded by the UKAID, the then Department for International Development (DfID) through the Transform Nutrition Research Consortium, and the Swedish International Development Cooperation Agency, Sweden
Carmichael, 2019	India	The Bill and Melinda Gates Foundation
Fottrell, 2013	Bangladesh	Big Lottery Fund International Strategic Grant. This study was supported with funds from a Wellcome Trust Strategic Award
Gupta, 2019	India	Swedish Agency for International Development Agency (SIDA)
Haider, 2000	Bangladesh	Swiss Agency for Development and Cooperation (SDC)
Jahan, 2014	Bangladesh	No funding information reported
Kim, 2018	Bangladesh	Bill & Melinda Gates Foundation, through Alive & Thrive, managed by FHI 360; additional financial support to the evaluation study was provided by the CGIAR Research Program on Agriculture for Nutrition and Health (A4NH), led by the International Food Policy Research Institute
Kumar, 2008	India	Bill & Melinda Gates Foundation
LeFevre, 2022	India	The Bill and Melinda Gates Foundation
Menon, 2016	Bangladesh	Bill & Melinda Gates Foundation
Modi, 2019	India	Indian Council of Medical Research
Nguyen, 2017	Bangladesh	Bill & Melinda Gates Foundation, the Canadian Department of Foreign Affairs, Trade, and Development
Nguyen, 2021	India	Bill & Melinda Gates Foundation, through Alive & Thrive (A&T)
Saville, 2018	Nepal	UKAID from Department for International Development South Asia Research Hub
Sikander, 2015	Pakistan	PRIDE, Pakistan (Primary Health Care Revitalisation, Integration, and Decentralisation in Earthquake Affected Areas), a project funded by the US Agency for International Development.
Talukder, 2017	Bangladesh	Bill & Melinda Gates Foundation to FHI 360, through the Alive & Thrive Small Grants Program managed by UC Davis.
Taneja, 2015; Bhandari, 2012	India	World Health Organization, Geneva (through umbrella grant from United States Agency for International Development); United Nations Children's Fund, New Delhi, and the Programme for Global Health and Vaccination Research of the Research Council of Norway.
Ullah, 2019	Bangladesh	Office of Health, Infectious Diseases, and Nutrition, Bureau for Global Health, U.S. Agency for International Development

10 PRISMA checklist

Table S9: PRISMA checklist

Section and Topic	Item #	Checklist item	Location where item is reported
**TITLE**			
Title	1	Identify the report as a systematic review.	Title
**ABSTRACT**			
Abstract	2	See the PRISMA 2020 for Abstracts checklist.	Abstract
**INTRODUCTION**			
Rationale	3	Describe the rationale for the review in the context of existing knowledge.	Paragraph 3
Objectives	4	Provide an explicit statement of the objective(s) or question(s) the review addresses.	Paragraph 3
**METHODS**			
Eligibility criteria	5	Specify the inclusion and exclusion criteria for the review and how studies were grouped for the syntheses.	Inclusion and exclusion criteria
Information sources	6	Specify all databases, registers, websites, organisations, reference lists and other sources searched or consulted to identify studies. Specify the date when each source was last searched or consulted.	Data source and search strategy
Search strategy	7	Present the full search strategies for all databases, registers and websites, including any filters and limits used.	Data source and search strategy, Supplementary file (Item 1)
Selection process	8	Specify the methods used to decide whether a study met the inclusion criteria of the review, including how many reviewers screened each record and each report retrieved, whether they worked independently, and if applicable, details of automation tools used in the process.	Selection process
Data collection process	9	Specify the methods used to collect data from reports, including how many reviewers collected data from each report, whether they worked independently, any processes for obtaining or confirming data from study investigators, and if applicable, details of automation tools used in the process.	Data extraction and covariates
Data items	10a	List and define all outcomes for which data were sought. Specify whether all results that were compatible with each outcome domain in each study were sought (e.g., for all measures, time points, analyses), and if not, the methods used to decide which results to collect.	Data extraction, Supplementary file (Item 5)
	10b	List and define all other variables for which data were sought (e.g., participant and intervention characteristics, funding sources). Describe any assumptions made about any missing or unclear information.	Supplementary file (Item 5)
Study risk of bias	11	Specify the methods used to assess risk of bias in the included studies, including details of the tool(s) used, how many reviewers assessed each study and whether they worked independently, and if applicable,	Risk of bias and strength of evidence

Section and Topic	Item #	Checklist item	Location where item is reported
assessment		details of automation tools used in the process.	
Effect measures	12	Specify for each outcome the effect measure(s) (e.g., risk ratio, mean difference) used in the synthesis or presentation of results.	Data synthesis and meta-analysis
Synthesis methods	13a	Describe the processes used to decide which studies were eligible for each synthesis (e.g., tabulating the study intervention characteristics and comparing against the planned groups for each synthesis (item #5)).	Data synthesis and meta-analysis
	13b	Describe any methods required to prepare the data for presentation or synthesis, such as handling of missing summary statistics, or data conversions.	Data synthesis and meta-analysis
	13c	Describe any methods used to tabulate or visually display results of individual studies and syntheses.	Data synthesis and meta-analysis
	13d	Describe any methods used to synthesize results and provide a rationale for the choice(s). If meta-analysis was performed, describe the model(s), method(s) to identify the presence and extent of statistical heterogeneity, and software package(s) used.	Data synthesis and meta-analysis
	13e	Describe any methods used to explore possible causes of heterogeneity among study results (e.g., subgroup analysis, meta-regression).	Data synthesis and meta-analysis
	13f	Describe any sensitivity analyses conducted to assess robustness of the synthesized results.	Data synthesis and meta-analysis
Reporting bias assessment	14	Describe any methods used to assess risk of bias due to missing results in a synthesis (arising from reporting biases).	Risk of bias and Strength of evidence
Certainty assessment	15	Describe any methods used to assess certainty (or confidence) in the body of evidence for an outcome.	Risk of bias and Strength of evidence
**RESULTS**			
Study selection	16a	Describe the results of the search and selection process, from the number of records identified in the search to the number of studies included in the review, ideally using a flow diagram.	Selection process and study characteristics, Figure 1
	16b	Cite studies that might appear to meet the inclusion criteria, but which were excluded, and explain why they were excluded.	Not applicable
Study characteristics	17	Cite each included study and present its characteristics.	Table 2, Supplementary file (Item 5)
Risk of bias in studies	18	Present assessments of risk of bias for each included study.	Risk of bias and Strength of evidence, Supplementary file (Item 8)
Results of individual studies	19	For all outcomes, present, for each study: (a) summary statistics for each group (where appropriate) and (b) an effect estimate and its precision (e.g. confidence/credible interval), ideally using structured tables or plots.	Table 3

Section and Topic	Item #	Checklist item	Location where item is reported
Results of syntheses	20a	For each synthesis, briefly summarise the characteristics and risk of bias among contributing studies.	Supplementary file (Item 8)
	20b	Present results of all statistical syntheses conducted. If meta-analysis was done, present for each the summary estimate and its precision (e.g., confidence/credible interval) and measures of statistical heterogeneity. If comparing groups, describe the direction of the effect.	Intervention effects and meta-analysis, Figure 2
	20c	Present results of all investigations of possible causes of heterogeneity among study results.	Intervention effects and meta-analysis, Supplementary file (Item 7)
	20d	Present results of all sensitivity analyses conducted to assess the robustness of the synthesized results.	Intervention effects and meta-analysis, Supplementary file (Item 3)
Reporting biases	21	Present assessments of risk of bias due to missing results (arising from reporting biases) for each synthesis assessed.	Risk of bias and strength of evidence, Supplementary file (Item 8)
Certainty of evidence	22	Present assessments of certainty (or confidence) in the body of evidence for each outcome assessed.	Risk of bias and strength of evidence, Supplementary file (Item 9)
**DISCUSSION**			
Discussion	23a	Provide a general interpretation of the results in the context of other evidence.	Paragraph 1
	23b	Discuss any limitations of the evidence included in the review.	Paragraph 6
	23c	Discuss any limitations of the review processes used.	Paragraph 6
	23d	Discuss implications of the results for practice, policy, and future research.	Paragraph 7
**OTHER INFORMATION**			
Registration and protocol	24a	Provide registration information for the review, including register name and registration number, or state that the review was not registered.	Methods (Conceptualization)
	24b	Indicate where the review protocol can be accessed, or state that a protocol was not prepared.	Methods (Conceptualization)
	24c	Describe and explain any amendments to information provided at registration or in the protocol.	Methods (Conceptualization)
Support	25	Describe sources of financial or non-financial support for the review, and the role of the funders or sponsors in the review.	Acknowledgement, Funding

Section and Topic	Item #	Checklist item	Location where item is reported
Competing interests	26	Declare any competing interests of review authors.	Competing interest
Availability of data, code and other materials	27	Report which of the following are publicly available and where they can be found: template data collection forms; data extracted from included studies; data used for all analyses; analytic code; any other materials used in the review.	Data availability statement
